# Anti-cancer activity of glucosamine through inhibition of N-linked glycosylation

**DOI:** 10.1186/1475-2867-14-45

**Published:** 2014-05-28

**Authors:** Viktor Chesnokov, Beata Gong, Chao Sun, Keiichi Itakura

**Affiliations:** 1 Department of Molecular and Cellular Biology, Beckman Research Institute of City of Hope National Medical Center, 1500 East Duarte Road, Duarte, California 91010, USA

**Keywords:** Glucosamine, N-linked glycosylation, IL-6, STAT3, Cancer

## Abstract

**Background:**

We have reported that the glucosamine suppressed the proliferation of the human prostate carcinoma cell line DU145 through inhibition of STAT3 signaling. DU145 cells autonomously express IL-6 and the IL-6/STAT3 signaling is activated. IL-6 receptor subunits are subject to N-glycosylation, a posttranslational modification which is important for protein stability and function. We speculated that the inhibition of STAT3 phosphorylation by glucosamine might be a functional consequence of the reduced N-glycosylation of gp130.

**Methods:**

The human prostate cancer cell lines DU145 and PC-3 and human melanoma cell line A2058 were used in this study. Glucosamine effects on N-glycosylation of glycoproteins were determined by Western blot analysis. IL-6 binding to DU145 cells was analyzed by flow cytometry. The cell proliferation suppression was investigated by colorimetric Janus green staining method.

**Results:**

In DU145 cells glucosamine reduced the N-glycosylation of gp130, decreased IL-6 binding to cells and impaired the phosphorylation of JAK2, SHP2 and STAT3. Glucosamine acts in a very similar manner to tunicamycin, an inhibitor of protein N-glycosylation. Glucosamine-mediated inhibition of N-glycosylation was neither protein- nor cell-specific. Sensitivity of DU145, A2058 and PC-3 cells to glucosamine-induced inhibition of N-glycosylation were well correlated to glucosamine cytotoxicity in these cells.

**Conclusion:**

Our results suggested that the glucosamine-induced global inhibition of protein N-glycosylation might be the basic mechanism underlying its multiple biochemical and cellular effects.

## Background

In the previous paper [1], we reported that glucosamine suppressed the proliferation and induced the death of human prostate carcinoma DU145 cells. This anti-cancer activity was associated with a decreased DNA synthesis, cell cycle arrest in G1 phase, and induction of apoptosis. We demonstrated for the first time that glucosamine inhibited the phosphorylation of signal transducer and activator of transcription (STAT) 3 at Tyr705 residue, thereby inhibiting the STAT3 activity in DU145 [1]. STAT3, a member of the STAT family, is activated by phosphorylation and mediates cellular responses to cytokines and growth factors [2]. Activated STAT3 promotes angiogenesis, inflammation, proliferation and survival of cancer cells and is frequently detected in numerous human tumors [3]. Suppression of the STAT3 signaling pathway [4-6] therefore represents a validated target for cancer therapy [3,7]. In DU145 cells, IL-6 is autonomously expressed and activates the IL-6/STAT3 signaling pathway by an autocrine mechanism [8]. IL-6 first binds to the two non-signaling α-receptor subunits (IL-6Rα/gp80) and the resultant complex recruits the two signal transducing receptor subunits (gp130) to form the functional ternary receptor complex. Janus kinases (JAKs) associated with gp130 are activated by phosphorylation and then STAT3 becomes activated by the phosphorylated JAKs [9].

Almost all receptors on the cell membrane are N-linked glycosylated proteins [10]. N-linked glycosylation (N-glycosylation) is a posttranslational modification; a preassembled core oligosaccharide moiety (glycan) is transferred to asparagine (N) residues at potential glycosylation sites of newly synthesized polypeptides and the attached N-linked glycans (N-glycans) are subjected to remodeling and branching [11]. N-glycosylation plays an important role for protein stability and functions [10,12]. Taking into account that gp130 is a highly N-glycosylated protein [13] and that glucosamine was proposed as an inhibitor of N-glycosylation [14], we speculated that the inhibition of STAT3 phosphorylation by glucosamine might be a functional consequence of the reduced N-glycosylation of gp130.

In this paper, we revealed that in DU145 cells glucosamine reduced the N-glycosylation of gp130, decreased IL-6 binding to cells and impaired the phosphorylation of JAK2 on Tyr1007/1008, SHP2 on Tyr542 and STAT3 on Tyr705 residues. Treatment with tunicamycin, an inhibitor of protein N-glycosylation [15], demonstrated all the effects of glucosamine supporting our speculation. We also showed that glucosamine-mediated inhibition of N-glycosylation was neither protein- nor cell-specific. Our results suggested that the glucosamine-induced global inhibition of protein N-glycosylation might be the basic mechanism underlying its multiple biochemical and cellular effects.

## Results

### Glucosamine reduces the molecular mass of gp130 in DU145 cells by the inhibition of co-translational N-glycosylation

**Figure 1:**
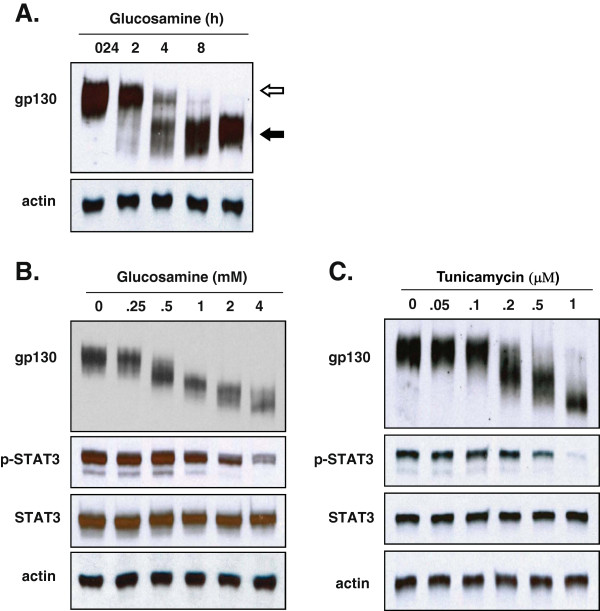
**Glucosamine reduced the molecular mass of gp130 and suppressed the phosphorylation of STAT3 in DU145 cells. (A)** Western blot analysis of the extracts from the cells cultured in the presence of 2 mM glucosamine for the indicated time (h). Whole-cell lysates were subjected to immunoblotting using antibodies specific for gp130 and actin (loading control). The *open arrow* indicates the molecular mass of gp130 without glucosamine treatment and the *filled arrow* indicates the reduced molecular mass of gp130 following the treatment. **(B)** Western blot analysis of cells cultured with indicated concentrations of glucosamine (mM) for 24 h. Whole-cell lysates were subjected to immunoblotting using antibodies specific for gp130, phospho (Tyr705)-STAT3 (p-STAT3), STAT3 and actin (loading control). **(C)** Western blot analysis of cells cultured with indicated concentrations of tunicamycin (μM) for 24 h. Whole-cell lysates were subjected to immunoblotting using the same antibodies as described for **B**. Each blot is a representative of three independent experiments.

**Figure 2:**
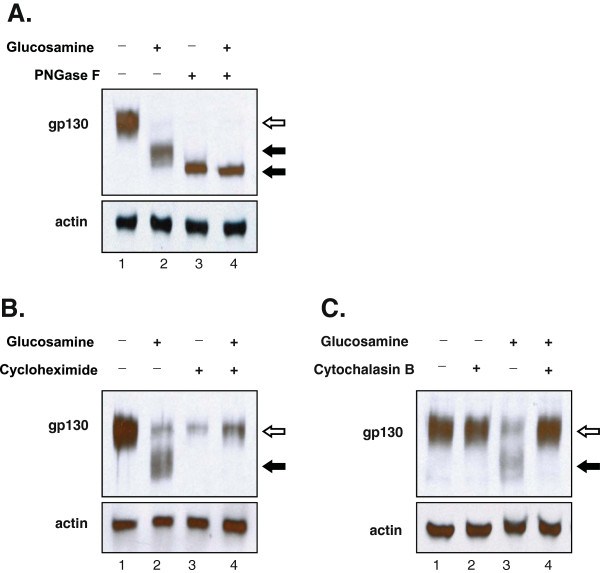
**Glucosamine inhibited co-translational N-glycosylation of gp130 and glucose transporter activity was essential for the inhibition. (A)** Western blot analysis of the whole-cell lysates treated *in vitro* with peptide-N-glycosidase F (PNGase F). DU145 cells cultured with or without 2 mM glucosamine for 24 h, and then whole-cell lysates were prepared and treated with or without peptide-N-glycosidase F (40 ug/ml) for 4 h at 37C followed by immunoblotting using antibodies specific for gp130 and actin (loading control). The *open arrow* indicates the molecular mass of N-glycosylated gp130 without glucosamine or PNGase F treatment and the *filled arrow* indicates reduced molecular mass of N-glycosylation deficient gp130. **(B)** Western blot analysis of cells treated with 2 mM glucosamine in the presence or absence of cycloheximide. DU145 cells cultured with or without 2 mM glucosamine for 4 h in the presence or absence of cycloheximide (100 μg/ml), and then the whole-cell extracts were prepared and subjected to immunoblotting using antibodies specific for gp130 and actin (loading control). The *open arrow* indicates the molecular mass of N-glycosylated gp130 and the *filled arrow* indicates the reduced molecular mass of N-glycosylation deficient gp130. **(C)** Western blot analysis of DU145 cells treated with glucosamine in the presence or absence of glucose transporter inhibitor cytochalasin B. Cells pre-incubated with 10 μM cytochalasin B for 30 min and then treated with 2 mM glucosamine for 4 h. The whole-cell extracts were prepared and subjected to immunoblotting using antibodies specific for gp130 and actin (loading control). The *open arrow* indicates the molecular mass of N-glycosylated gp130 and the *filled arrow* indicates the reduced molecular mass of N-glycosylation deficient gp130. Each blot is a representative of three independent experiments.

To prove our hypothesis that the inhibition of STAT3 phosphorylation by glucosamine might be a functional consequence of the reduced N-glycosylation of gp130, we first studied the effect of glucosamine on the gp130 expression in DU145 cells by Western blot analysis. The treatment of DU145 cells with 2 mM glucosamine resulted in a time-dependent accumulation of gp130 proteins with a reduced molecular mass of 100-110 kDa (marked with *filled arrows*) and the proteins with an original mass (marked with *open arrow*) became undetectable after 24 h (Figure 1A). However, the molecular mass of actin was unaffected. The extent of gp130 molecular mass reduction was in proportional to glucosamine concentrations (0.25-4 mM) for a 24 h treatment. At 0.25 mM, a slight reduction of the molecular mass was observed and at 4 mM, the original gp130 proteins completely disappeared (Figure 1B, the first row). In parallel to the reduction of the gp130 molecular mass, the phosphorylation of STAT3 at Tyr705 residue gradually decreased (the second row). It is noteworthy that the same range of glucosamine concentrations (0.5 – 4 mM) in this experiment proportionally inhibited the proliferation of DU145 cells as published [1]. The observed reduction of gp130 molecular mass could occur as a result of either the loss of post-translational modifications such as N-glycosylation, proteolytic cleavage of translated products, premature termination of translation, or a combination of these processes. Gp130 has 11 potential N-glycosylation sites and 9 of them are actually N-glycosylated [13]. To investigate the mechanism underlying the glucosamine-induced reduction of gp130 molecular mass, DU145 cells were treated with tunicamycin, a proven inhibitor of N-glycosylation [15]. As shown in Figure 1C, the tunicamycin treatment resulted in a gradual reduction of the molecular mass of gp130 in a concentration-dependent manner similar to glucosamine, albeit using about one thousandth of the concentration. The reduction of the molecular mass was accompanied by the suppression of STAT3 phosphorylation at Tyr705. These results showed that glucosamine precisely reproduced the pattern of the tunicamycin treatment (Figures 1B, C) and suggested that the glucosamine-induced reduction of gp130 molecular mass was caused by the inhibition of N-glycosylation. To exclude the possibility of proteolytic cleavage or premature translation termination, extracts from DU145 cells cultured in the presence or absence of glucosamine were treated *in vitro* with N-glycanase F (PNGase F), which removes N-glycans from proteins regardless of the levels of their initial N-glycosylation (Figure 2A). Incubation of cell extracts derived from the untreated cells (lane 1, original gp130, *open arrow*) or the glucosamine- treated cells (lane 2, gp130 with reduced molecular mass, *filled arrow*) with PNGase F generated protein products with the identical molecular mass (lane 3 and 4, *filled arrow*). These data proved that neither proteolytic cleavage nor premature translation termination was involved in the reduction of gp130 molecular mass in the treatment with glucosamine. Furthermore, the treatment of DU145 cells with glucosamine in the presence of cycloheximide, a translational inhibitor, completely abolished the appearance of gp130 proteins with lower molecular mass (Figure 2B, lane 4). The data indicate that glucosamine does not remove N-glycans from the mature gp130, but inhibits N-glycosylation of the newly synthesized protein. Finally, as shown in Figure 2C, cytochalasin B, an inhibitor of glucose transporters [16], blocked the glucosamine-induced reduction of the gp130 molecular mass (lane 4) suggesting that cells uptake glucosamine by glucose transporters. Altogether, these results clearly demonstrate that glucosamine is transported into DU145 cells via glucose transporters and suppresses co-translational N-glycosylation to produce gp130 proteins with a lower molecular mass.

### Glucosamine-induced inhibition of N-glycosylation of gp130 represses the IL6/JAK/STAT3 signaling in DU145 cells

**Figure 3:**
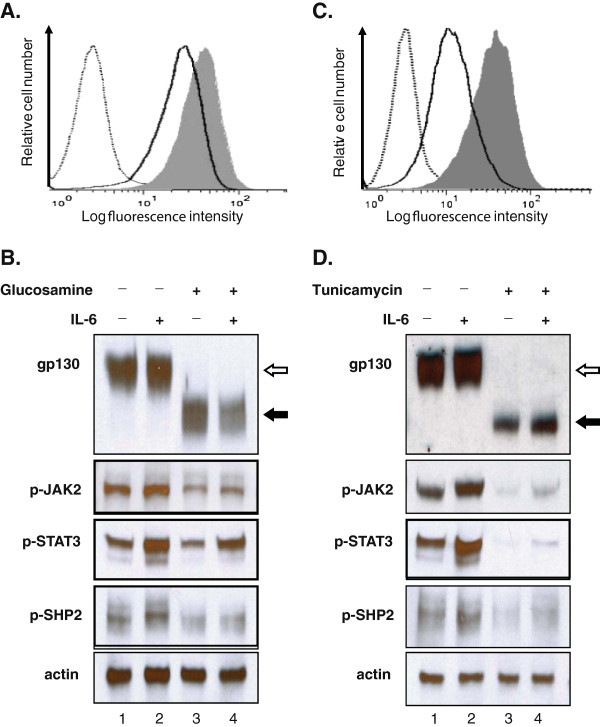
**Glucosamine suppressed the IL-6/JAK/STAT3 signaling pathway in DU145 cells. (A)** Glucosamine decreased IL-6 binding to DU145 cells. Cells cultured with or without 2 mM glucosamine for 24 h, and then incubated with a fluorescently labeled IL-6 followed by FACS analysis. Gray histogram represents IL-6 binding to cells without glucosamine treatment, bold line histogram represents IL-6 binding to glucosamine-treated cells and dotted line histogram represents negative control, cells without fluorescently labeled IL-6. **(B)** Western blot analysis of cells cultured with or without glucosamine following the IL-6 treatment. Cells cultured in serum free medium with or without 2 mM glucosamine for 24 h and then treated with 2 ng/ml IL-6 for 15 min. Whole-cell lysates were subjected to immunoblotting using antibodies specific for gp130, phospho (Tyr1007/1008)-JAK2 (p-JAK2), phospho (Tyr705)-STAT3 (p-STAT3), phospho (Tyr542)-SHP2 (p-SHP2) and actin (loading control). The *open arrow* indicates N-glycosylated gp130 and the *filled arrow* indicates N-glycosylation deficient gp130. **(C)** Tunicamycin decreases IL-6 binding to DU145 cells. Cells were cultured with or without 1 μg/ml tunicamycin for 24 h and then incubated with a fluorescently labeled IL-6 following FACS analysis. Gray histogram represents IL-6 binding to cells without tunicamycin treatment, bold line histogram represents IL-6 binding to tunicamycin-treated cells and dotted line histogram represents negative control, cells without fluorescently labeled IL-6. **(D)** Western blot analysis of cells cultured with or without tunicamycin followed by the IL-6 treatment. Cells cultured in serum free medium with or without 1 μg/ml tunicamycin for 24 h and then treated with 2 ng/ml IL-6 for 15 min. Whole-cell lysates were analyzed and data were presented in the same way as for glucosamine (Figure 3B). The binding assays and each blot are a representative of three independent experiments.

To determine whether the deficiency in N-glycosylation has any effects on the activity of the gp130-associated IL-6/JAK/STAT3 signaling [9], we carried out the following investigations. First, we studied IL-6 binding to DU145 cells in the presence and absence of glucosamine. Cells were pre-treated with glucosamine (2 mM for 24 h) and IL-6 binding to the cells were analyzed. The flow cytometry binding assays revealed that the preincubation of DU145 cells with glucosamine considerably shifted the intensity of IL-6 fluorescence to a lower side indicating less binding of IL-6 to cells as compared to the untreated control (Figure 3A). Next, we analyzed the tyrosine phosphorylation of the down-stream signaling molecules of IL-6 receptor including JAK2, STAT3 and SHP2. DU145 cells secrete IL-6, which stimulates the phosphorylation of these molecules via an autocrine fashion [8]. As shown in Figure 3B, basal levels of the phosphorylated JAK2 (Tyr1007/1008, p-JAK2), STAT3 (Tyr705, p-STAT3) and SHP2 (Tyr542, p-SHP2) were detected (lane 1), and exogenous IL-6 (2 ng/ml, 15 min) further increased the tyrosine phosphorylation of these signaling proteins (lane 2). Glucosamine treatment decreased the levels of both the basal (lane 1 vs. 3) and IL-6-induced (lane 2 vs. 4) tyrosine phosphorylation of JAK2, STAT3 and SHP2. These results demonstrated that glucosamine prevented IL-6 from binding to cells and suppressed the multiple steps of the IL-6/JAK/STAT3 signaling pathway. To confirm further that the inhibition of N-glycosylation could be responsible for the decrease of IL-6 binding to cells and the impairing of the IL-6/JAK/STAT3 signaling, DU145 cells were treated with tunicamycin in a similar way to glucosamine. The results showed that tunicamycin reduced IL-6 binding to cells (Figure 3C) and impaired both the basal and IL-6-induced tyrosine phosphorylation of JAK2, STAT3 and SHP2 (Figure 3D, lane 3 and 4). We concluded that the glucosamine-induced inhibition of gp130 N-glycosylation reduced IL-6 binding to cells to suppress the IL-6/JAK/STAT3 signaling pathway.

### Glucosamine affects on multiple N-glycosylated proteins

**Figure 4:**
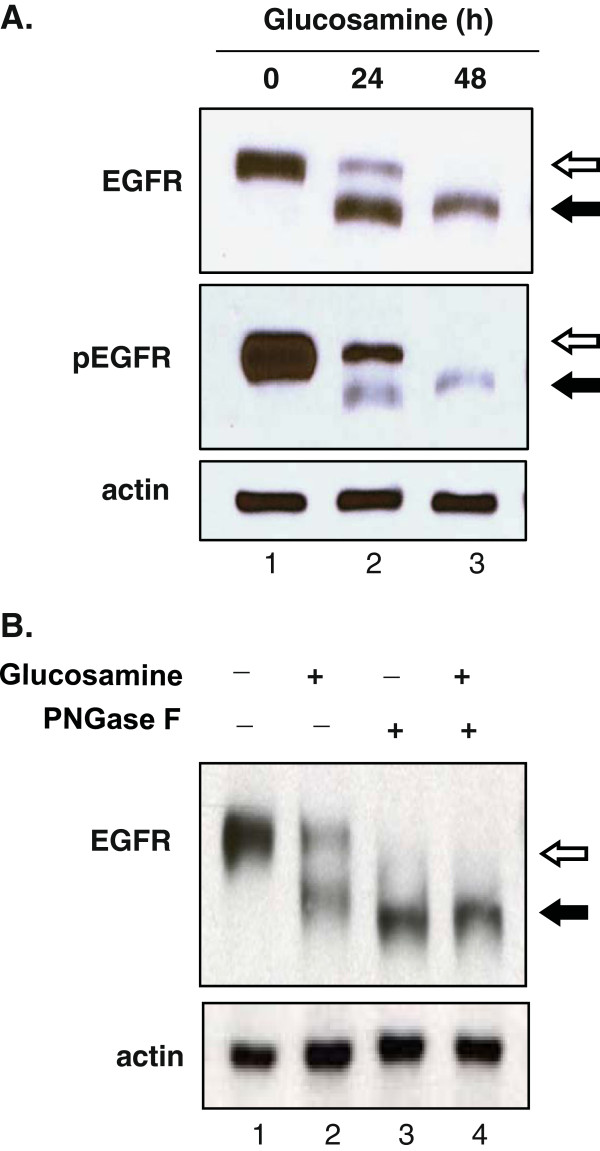
**Glucosamine inhibited N-glycosylation and auto-phosphorylation of EGFR in DU145 cells. (A)** Western blot analysis of cells cultured in the presence of 2 mM glucosamine for 24 or 48 h. Whole-cell lysates were subjected to immunoblotting using antibodies specific for EGFR, phospho (Tyr845)-EGFR (pEGFR) and actin (loading control). The *open arrow* indicates the N-glycosylated receptor and its phosphorylated form, and the *filled arrow* indicates the N-glycosylation deficient receptor and its phosphorylated form. **(B)** Western blot analysis of the whole-cell lysates treated *in vitro* with peptide-N-glycosidase F (PNGase F). DU145 cells cultured with or without 2 mM glucosamine for 24 h, and then the whole-cell lysates were prepared and treated with or without peptide-N-glycosidase F (40 ug/ml) for 4 h at 37C followed by immunoblotting using antibodies specific for EGFR and actin (loading control). The *open arrow* indicates the molecular mass of N-glycosylated EGFR and the *filled arrow* indicates the reduced molecular mass of N-glycosylation deficient EGFR. Each blot is a representative of three independent experiments.

**Figure 5:**
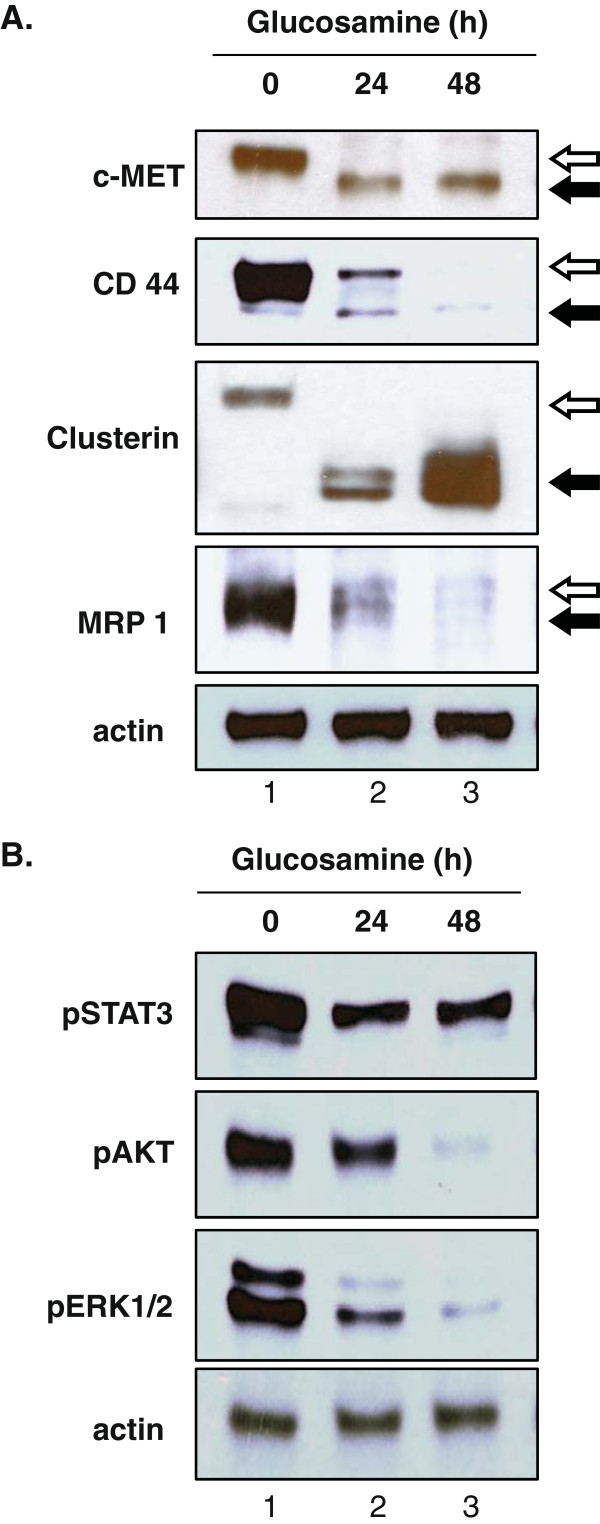
**Glucosamine reduced the molecular mass of various glycoproteins by inhibiting N-glycosylation and suppressed the multiple signaling pathways in DU145 cells. (A)** Western blot analysis of cells cultured in the presence of 2 mM glucosamine for 24 or 48 h. Whole-cell lysates were prepared and subjected to immunoblotting using antibodies specific for c-MET, CD 44, clusterin, MRP1 and actin (loading control). The *open arrow* indicates the molecular mass of proteins without glucosamine treatment and the *filled arrow* indicates the reduced molecular mass of protein following the treatment. **(B)** Western blot analysis of the phosphorylated (active) STAT, AKT and ERK1/2 proteins after glucosamine treatment. The whole-cell lysates employed in Figure 5A were subjected to immunoblotting using antibodies specific for phospho (Tyr705)-STAT3 (pSTAT3), phospho (Ser473)-AKT (pAKT), phospho (Thr202/Tyr204)-ERK1/2 (pERK1/2) and actin (loading control). Each blot is a representative of three independent experiments.

To demonstrate that the glucosamine-induced inhibition of N-glycosylation was not limited to gp130, we analyzed the effect of glucosamine on the other N-glycosylated protein EGFR in DU145 cells. The 170 kDa human EGFR has 11 potential N-glycosylation sites, 8 of them are fully glycosylated, and one site is only partially glycosylated [17]. As shown in the upper panel of Figure 4A, treatment of cells with glucosamine (2 mM) resulted in the reduction of the expression level of the 170 kDa EGFR protein (marked with *open arrows*) and the appearance of proteins with a lower molecular mass (marked with *filled arrow*) in a time dependent manner as it was observed for gp130 (Figure 1A). We verified that neither proteolytic cleavage nor premature translation termination caused the reduction of the molecular mass by glucosamine (Figure 4B). Incubation of extracts from both the untreated (lane 1) and glucosamine treated cells (lane 2) with PNGase F gave de-N-glycosylated EGFR proteins with an identical molecular mass (lanes 3 and 4). Since the effect on EGFR was identical to that on gp130 (Figures 1A and 2A), we concluded that glucosamine suppresses N-glycosylation of EGFR. EGFR is a tyrosine kinase receptor which is activated by autophosphorylation at Tyr845 residue upon ligand binding [18] and we examined whether the activity was altered by de-N-glycosylation. As shown on Figure 4A, the non-treated 170 kDa form of EGFR was strongly phosphorylated at Tyr 845 residue in the cells cultured with 10% serum (lane 1 marked with *open arrow*), whereas EGFR with a lower molecular mass that was deficient in N-glycosylation was weakly phosphorylated at this residue (lanes 2 and 3 marked with *filled arrow*). To further confirm that glucosamine inhibited global protein N-glycosylation, in addition to gp130 and EGFR, we analyzed four other N-glycosylated proteins including C-MET, clusterin, CD44 and MRP1 in DU145 cells. Western blot analysis of cell extracts revealed the time dependent increase of the mobility of those proteins in the presence of glucosamine (Figure 5A). Taken all the results together, we concluded that in DU145 cells glucosamine depleted a pool of N-glycosylated proteins by suppressing N-glycosylation of the newly synthesized proteins. To address the functional consequence of the glucosamine-induced inhibition of global protein N-glycosylation, we examined the phosphorylation (activity) of three important signaling proteins in cancer cells, STAT3 (Tyr705), AKT (Ser473) and ERK1/2 (Thr202/Tyr204) that were activated by membrane N-glycosylated receptors. We found that phosphorylation of all these proteins in cells cultured with serum (10% FBS) was suppressed by the treatment of 2 mM glucosamine in a time dependent manner (Figure 5B, lanes 2 and 3). These data suggest that suppression of multiple signaling pathways may contribute to the glucosamine anti-cancer activity.

### Sensitivity of cells to glucosamine-induced inhibition of N-glycosylation is correlated to glucosamine anti-cancer activity

**Figure 6:**
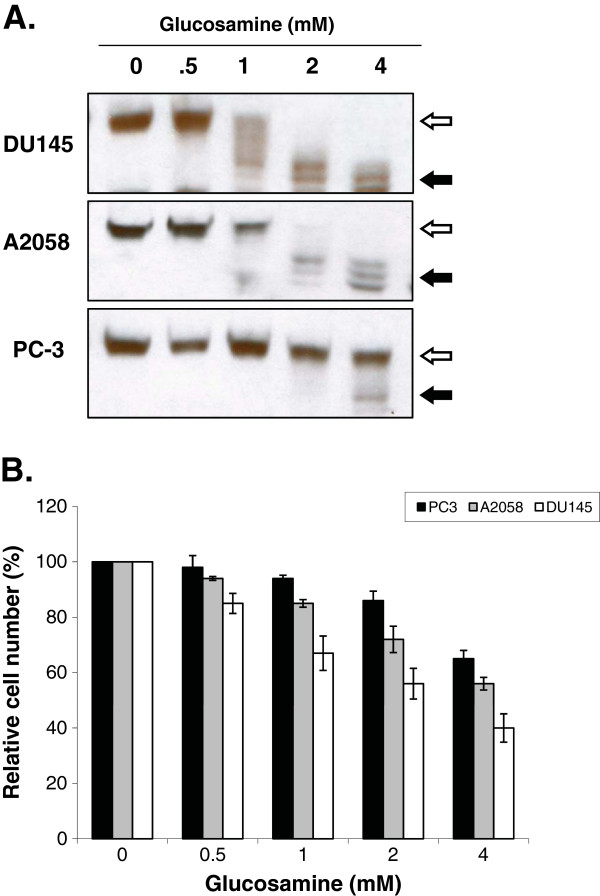
**The sensitivity of cells to the inhibition of N-glycosylation by glucosamine was correlated to glucosamine toxicity. (A)** Western blot analysis of DU145, A2058 and PC-3 cells cultured with the indicated concentrations of glucosamine (mM) for 4 h. Whole-cell lysates were subjected to immunoblotting using antibodies specific for clusterin as N-glycosylation marker. The *open arrow* indicates the molecular mass of N-glycosylated clusterin and the *filled arrow* indicates N-glycosylation deficient clusterin. Each blot is a representative of three independent experiments. **(B)** The viability of DU145, A2058 and PC-3 cells cultured in the presence of the indicated concentration (mM) of glucosamine for 48 h. The viability of cells was measured by cell counting using the colorimetric Janus green whole-cell stain method. The results were present as mean+/-SD of four independent experiments.

To examine whether the inhibition of N-glycosylation is cell-specific, we compared the sensitivity of two human prostate carcinoma DU145 and PC-3 cells and human melanoma A2058 cells to glucosamine. The cells were cultured in 0.5, 1, 2, or 4 mM glucosamine for 4 h, and the inhibition of N-glycosylation was assessed by Western blot analysis of clusterin. As shown in Figure 6A, an increased mobility of clusterin with the inhibition of N-glycosylation was detected at 1 mM, 2 mM and 4 mM in DU145 cells, A2058 cells and PC-3 cells, respectively. Western blot analysis of the others N-glycosylated proteins (gp130 and EGFR) in the respective cells produced the results identical to clusterin (data not shown). These data indicate that DU145 cells were the most sensitive to the glucosamine-induced inhibition of N-glycosylation, followed by A2058 cells, and PC-3 cells were the least sensitive. To investigate whether the inhibition of N-glycosylation correlated to the anti-cancer activity of glucosamine, we carried out cell viability assays of these cells under the same conditions. As shown in Figure 6B, the toxicity of glucosamine to DU145, A2058 and PC-3 cells correlated directly with the inhibition of N-glycosylation. These results suggest that the glucosamine-induced inhibition of protein N-glycosylation might be one of the basic mechanisms that accounts for the glucosamine anti-cancer activity.

## Discussion

Glucosamine, an amino monosaccharide has been widely used as a dietary supplement to relieve discomfort of rheumatoid arthritis or osteoarthritis for more than fifty years [19]. Glucosamine has also been reported as an inhibitor of tumor growth both *in vivo*[20] and *in vitro*[21]. However, the mechanism for the anti-cancer activity is still not fully understood to explore the potentials of glucosamine as an anti-cancer agent. Previously [1], we have proposed our hypothesis that glucosamine could inhibit the N-glycosylation of IL-6 receptor in human prostate carcinoma DU145 cells, thereby reducing the activity of the IL-6/JAK/STAT3 signaling pathway. This pathway is activated and contributes to carcinogenesis in many different tumors. This study focused on proving our hypothesis and examining the mechanism by which the STAT3 activity is suppressed by glucosamine. When DU145 cells were treated with glucosamine, Western blot analysis showed that several gp130 proteins with a lower molecular mass appeared in a time and dose dependent manner (Figure 1). In the following experiments, we demonstrated that digestion of the extracts from the untreated and glucosamine-treated DU145 cells with peptide-N-glycosidase F (PNGase F), which removed all N-linked glycans [22], provided proteins with the same mobility on Western blot gels regardless of the mobility of the proteins before the digestion. These results indicated that gp130 proteins with a lower molecular mass had less N-glycosylated sites and therefore, were not truncated products of translation step. Furthermore, the glucosamine treatment of DU145 cells in the presence of cycloheximide did not produce any gp130 proteins with a lower molecular mass suggesting that glucosamine inhibited N-glycosylation of the newly synthesized protein but did not remove N-glycans from the mature gp130 (Figure 2). IL-6 receptor is composed of four subunits, two gp80 subunits containing the IL-6 binding domain and two gp130 subunits containing the signal transducing domain. Flow cytometry binding assays showed that IL-6 bound weakly to cells treated with glucosamine. We speculated that the gp130 subunit deficient in N-glycosylation could not form the intact IL-6 receptor with the gp80 subunit and thereby the binding of IL-6 to cells was suppressed to reduce the signal transduction. It was shown that N-glycosylation-deficient gp130 after tunicamycin treatment lost the ability to form a heterodimer with the leukemia inhibitory factor (LIF) binding subunit, and lost the signal transduction in response to LIF in neuroepithelial cells [23]. To further confirm that glucosamine acts in a similar manner to tunicamycin, we carried out the same experiments described above using tunicamycin in place of glucosamine. Tunicamycin is a proven inhibitor of protein N-glycosylation which blocks the transfer of N-acetylglucosamine to dolichol in the first step of oligosaccharide synthesis [15]. Indeed, tunicamycin lowered the molecular mass of the normally expressed gp130, suppressed IL-6 binding to cells and reduced the activity of the IL-6/JAK/STAT3 signaling pathway in a dose dependent manner like glucosamine (Figure 1C, 3). Obviously, various proteins are N-glycosylated and the modification of proteins by N-glycosylation is not specific to gp130. To confirm that these N-glycosylated proteins are substrates for glucosamine, we carried out the Western blot analysis of several glycoproteins, EGFR, c-MET, CD44, clusterin and MRP1 after the glucosamine treatment. In addition to gp130, the treatment produced these glycoproteins with a lower molecular mass in a different extent (Figure 5A). Since most of cell-surface receptors are N-glycosylated [10], it was expected that multiple signaling pathways activated from these receptors were affected by glucosamine. Indeed, Western blot analysis showed that in addition to STAT3, the phosphorylation (activity) of other proteins playing key signaling roles in cancer cells, AKT and ERK1/2, were suppressed after the treatment with glucosamine in DU145 cells (Figure 5B). These results suggest that glucosamine-induced inhibition of global protein N-glycosylation might be the basic mechanism of multiple biochemical and cellular events of its anti-cancer action. In fact, the sensitivity of different cells to glucosamine measured by de-N-glycosylation of clusterin was in a good agreement with their viability (Figure 6). As it was proposed, targeting multiple signaling pathways by disrupting posttranslational N-glycosylation in a combination with other agents might be an effective strategy for cancer therapy [24]. For example, tunicamycin treatment enhanced the susceptibility of human non-small cell lung cancer cells to EGFR tyrosine kinase inhibitor erlotinib [25], and also stimulated TRAIL–induced apoptosis in human prostate cancer PC-3 cells [26]. Unfortunately, tunicamycin is too toxic to use for the human treatment [27]. Alternatively, glucosamine could be a candidate for clinical use as an inhibitor of posttranslational N-glycosylation because of its low toxicity to the normal cells and enhanced uptaken by tumor cells due to “Warburg effect” [28]. We will investigate this possibility and study the mechanism underlying inhibition of N-glycosylation by glucosamine.

## Conclusions

Current study found that, in human prostate carcinoma cell line DU145, glucosamine reduced N-glycosylation of IL-6 receptor subunit gp130 by inhibiting co-translational N-glycosylation, resulting in less IL-6 binding to cells and less phosphorylation (activation) of down-stream JAK2 and STAT3 proteins. Our results demonstrated that glucosamine-mediated inhibition of N-glycosylation was neither protein- nor cell-specific. Glucosamine induced global inhibition of protein N-glycosylation in all three cancer cell lines which were used in this study. Several important signals, such as STAT3, AKT and ERK1/2, were activated by N-glycosylated membrane receptors. The treatment of glucosamine led to the suppression of these signals, suggesting that the glucosamine-induced inhibition of N-glycosylation might be the basic mechanism underlying its biochemical and cellular effects.

## Methods

### Cell culture, chemical compounds and biological reagents

The human prostate cancer DU145 and PC3 cells and human melanoma A2058 cells were obtained from the American Type Culture Collection. Cells were cultured in RPMI medium 1640 supplemented with glutamine, essential amino acids, 10% fetal bovine serum and antibiotics (100 units/ml penicillin G and 100 ug/ml streptomycin sulfates). Cells were incubated at 37°C in 5% CO2, and the medium was changed every 3-4 days. Cells were passaged at 70% confluent using trypsin/EDTA. Treatments of cells with D-glucosamine hydrochloride, tunicamycin, cycloheximide, cytochalasin B and interleukin-6 (IL-6) were performed in 6-well plates (Corning, Inc., Corning, NY). D-glucosamine hydrochloride, tunicamycin, cycloheximide, propidium iodide and cytochalasin B were purchased from Sigma Chemical Co., (St. Louis, MO; G-1514, T-7765, C-4859, C-6762, P-1607, and C2743). IL-6 and Human IL-6 Flow cytometry Kit were purchased from R & D Systems (Minneapolis, MN; 206-IL, NF600). Peptide: N-glycanase F (PNGase F) was purchased from New England Biolabs (Ipswich, MA; PO704). Antibodies used for immunoblot (Western) analysis included anti-gp130, anti-clusterin (Millipore, Danvers, MA; 06-291, 05-354), anti-STAT3, anti-phospho (Tyr705)-STAT3, anti-phospho (Tyr1007/1008)-JAK2, anti-phospho (Tyr542)-SHP2, anti-EGFR, anti-phospho (Tyr845)-EGFR, anti-phospho (Ser473)-AKT, anti-phospho (Thr202/Tyr204)-ERK1/2, anti-c-Met, anti-CD44, anti-mouse IgG HRP-linked and anti-rat IgG HRP-linked from Cell Signaling Technology (Beverly, MA; 9132, 9145, 3771, 3751, 2232, 2231, 4060, 4370, 3127, 5640, 7076, 7077), anti-actin and anti-MRP1 from Santa Cruz Biotechnology (Santa Cruz, CA; 1615, 59607). Additionally, Janus Green whole-cell stain was purchased from ThermoScientific-Pierce (Rockford, IL; 62203).

### Western blotting

After removing the culture medium cells in 6-well plates were washed with 1× PBS and then lysed in the wells with 0.1 ml of RIPA lysis and extraction buffer (25 mM Tris-HCl, 150 mM NaCl, 1% NP-40, 1% Sodium deoxycholate, 0.1% SDS, pH 7.6, (G-Bioscience, St. Louis, MO; 786-489,) supplemented with protease and phosphatase inhibitor cocktail (ThermoScientific-Pierce, Rockford, IL; 78440) for 15 min at 4°C. Lysates were then transferred to 1.5 ml microcentrifuge tubes, vortexed at maximum speed for 15 sec to shear DNA and centrifuged at 12000 g for 10 min at 4°C. Supernatants were quantified for protein concentrations by BCA protein assay kit (ThermoScientific-Pierce, Rockford, IL; 23227) and stored at -80°C in aliquots or used immediately for SDS-PAGE. The samples for SDS-PAGE were prepared by incubating with 4% SDS at 37°C for 30 min for solubilizing N-glycosylation deficient proteins and then boiling in the sample buffer (50 mM Tris-HCI pH 6.8, 2% SDS, 10% glycerol, 1% β-mercaptoethanol, 12.5 mM EDTA, 0.02% bromophenol blue) for 5 min. Equal amounts of proteins were then loaded onto 8% precast SDS-PAGE gels (ThermoScientific-Pierce, Rockford, IL; 25200). Immunoblotting was performed after the electrophoretic transfer of proteins onto nitrocellulose membrane (Bio-Rad Laboratories, Hercules, CA; 162-0115). The proteins were detected using protein-specific first antibodies, horseradish peroxidase-conjugated second antibodies and chemiluminescent detection reagent (Denville Scientific, Metuchen, NJ; E2500) according to the manufacturer’s conditions. Protein sizes on nitrocellulose membranes were detected by protein markers (Bio-Rad Laboratories, Hercules, CA; 161-0374). To reuse, nitrocellulose membranes were washed with western blot stripping buffer (ThermoScientific-Pierce, Rockford, IL; 46430) and blocked again.

### Peptide: N-glycanase F (PNGase F) treatment

To remove N-glycans from glycoproteins *in vitro* aliquots of whole cell extracts in RIPA buffer were incubated with Peptide:N-glycanase F (40 μg/μl) at 37°C for 4 h and then stored at -80°C or used immediately for Western blot analysis.

### Evaluation of IL-6 binding to DU145 cells by flow cytometry

The Human IL-6 Flow Cytometry Kit (R & D Systems) was utilized for analysis of IL-6 binding according to the manufacturer’s protocol with slight modifications. Briefly, DU145 cells were harvested by the treatment with 1 mM EDTA for 3-5 min, washed two times with phosphate-buffered saline (PBS), resuspended in PBS (4 × 10^6^/ml) and followed by the incubation of 50 μl of cells for 1 hour at 4°C with either 20 μl of biotinylated IL-6 or 20 μl of biotinylated negative control reagent. 20ul of Avidin-fluorescein was then added to each set of cells and incubation was continued for a further 30 minutes at 4°C. Cells were then washed two times with the supplied wash buffer, resuspended with 300 μl of wash buffer containing 10 ug/ml propidium iodide (PI) to identify dead cells and examined by flow cytometry. A total of 40000 cells were counted for each analysis. PI-positive/dead cells were subsequently excluded from the IL-6 binding examination. Fluorescence was measured using the CyAn System with Summit software (Beckman Coulter, Brea, CA).

### Cell growth suppression analysis

DU145, PC-3 or A2058 cells were plated in 96-well plates in a complete culture medium with or without different amount of glucosamine. Two days later, the relative cell numbers per well were determined by the colorimetric Janus green whole-cell stain method. Briefly, culture medium was removed from cell layers, then cells were fixed with 4% formaldehyde (Thermo Scientific, Rockford, IL; 28906) for 30 min at room temperature, and followed by vacuum aspiration of the fixative. Subsequently, fixed cell layers were stained with Janus Green Whole-Cell Stain solution (Thermo Scientific, Rockford, IL; 62203) for 5 min at room temperature. Then the excess stain was removed by vacuum aspiration and cells were washed 4 times with water. The dyes associated with cell layers were then eluted by the addition of 100 μl of 0.5 N HCl per well and shaking the plates at room temperature for 1 h. The colored dye solutions were read in a microplate reader (ASYS UVM340 Microplate Reader, Cambridge UK) at 615 nm. Results were expressed as a percentage of the control without glucosamine. Data were represented as mean +/- standard deviation (SD) from quadruplicate wells. Each cell line was analyzed in three independent experiments.

## Competing interests

The authors do not have any financial or personal relationships with other people or organizations that could inappropriately influence the work described in this manuscript.

## Authors’ contributions

VC conceived the idea, designed the study and performed the most of experiments. BG and CS involved in the experiments and manuscript editing. KI coordinated and supervised the study and edited the manuscript. All authors have read and approved the final manuscript.
